# Cell Death in Pulmonary Arterial Hypertension

**DOI:** 10.7150/ijms.93902

**Published:** 2024-07-14

**Authors:** Xia Li, JunLan Tan, JiaJing Wan, BeiBei Cheng, Yu-Hong Wang, Aiguo Dai

**Affiliations:** 1 Hunan Academy of Chinese Medicine, Changsha 410208, Hunan, People's Republic of China.; 2 Department of Respiratory Diseases, Medical School, Hunan University of Chinese Medicine, Changsha 410208, Hunan, People's Republic of China.; 3 Hunan Provincial Key Laboratory of Vascular Biology and Translational Medicine, Changsha 410208, Hunan, People's Republic of China.; 4 Science and Technology Innovation Center, Hunan University of Chinese Medicine, Changsha 410208, China.

**Keywords:** pulmonary arterial hypertension, pulmonary artery smooth muscle cells, pulmonary artery endothelial cells, cell death

## Abstract

Pulmonary arterial hypertension (PAH) is a severe pulmonary vascular disease characterized by increased pulmonary vascular resistance because of vascular remodeling and vasoconstriction. Subsequently, PAH leads to right ventricular hypertrophy and heart failure. Cell death mechanisms play a significant role in development and tissue homeostasis, and regulate the balance between cell proliferation and differentiation. Several basic and clinical studies have demonstrated that multiple mechanisms of cell death, including pyroptosis, apoptosis, autophagy, ferroptosis, anoikis, parthanatos, and senescence, are closely linked with the pathogenesis of PAH. This review summarizes different cell death mechanisms involved in the death of pulmonary artery smooth muscle cells (PASMCs) and pulmonary artery endothelial cells (PAECs), the primary target cells in PAH. This review summarizes the role of these cell death mechanisms, associated signaling pathways, unique effector molecules, and various pro-survival or reprogramming mechanisms. The aim of this review is to summarize the currently known molecular mechanisms underlying PAH. Further investigations of the cell death mechanisms may unravel new avenues for the prevention and treatment of PAH.

## Introduction

Pulmonary hypertension (PAH) is a complex disease characterized by a progressive increase in the pulmonary vascular resistance (PVR) and the pulmonary artery pressure (PAP). Vascular remodeling is a central feature of the pathophysiology of PAH and is characterized by structural and functional changes in the pulmonary artery wall. This leads to increased muscularization of the pulmonary artery wall and the peripheral non-muscular vessels of the respiratory acinar artery, and the formation of neointima and plexiform lesions. The global prevalence of PAH is 93 cases per 1 million subjects with a male-to-female prevalence ratio of 1:2.3 and a 3-year survival rate of only 55-65% 1,2. The interplay between various cell death mechanisms and other cellular processes drives the complex etiology, pathogenesis, and progression of PAH. This review describes various cell death mechanisms, specific cell death effector molecules, and associated signaling pathways in PAH. It also highlights the pro-survival or reprogramming mechanisms for circumventing cell death. The aim of this review is also to highlight future directions in this field and identify novel therapeutic targets for the prevention and treatment of PAH (Table 1).

## Milestones of the discovery of the role of cell death mechanisms in PAH

**Figure 1:**
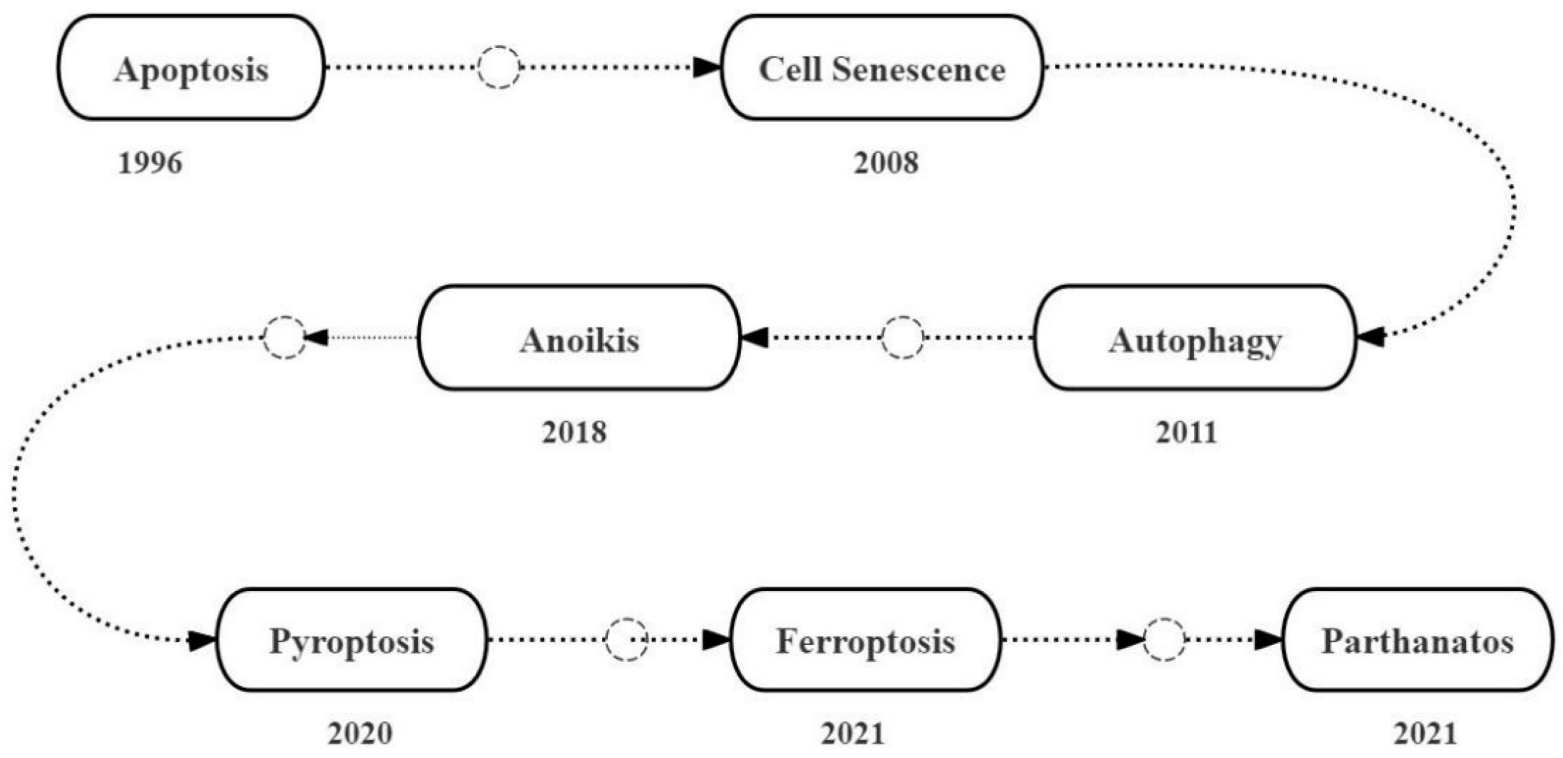
Milestones of the discovery of the roles of multiple cell death mechanisms in PAH.

In 1996, Jones and Rabinovitch reported the occurrence of endothelial cell apoptosis during the early stages of monocrotaline (MCT)-induced pulmonary arterial hypertension (PAH) in the adult Sprague-Dawley rats 3. In 2008, Marsboom *et al.* reported that chronic hypoxia exacerbated PAH by inducing senescence of the circulating endothelial progenitor cells (EPCs) in the C57Bl6/N mice 4. In 2011, Lee *et al.* reported that autophagy was upregulated in the lung tissues of patients with idiopathic pulmonary hypertension (IPAH), *in vitro* hypoxia-treated human primary endothelial cells (PAECs) and smooth muscle cells (PASMCs), and the chronic hypoxia-induced PAH model mice 5. In 2018, Chen *et al.* reported that PASMCs underwent anoikis, a novel type of apoptosis, because of detachment of PASMCs from the extracellular matrix due to reduced levels of integrin α5 and β1 during PAH 6. In 2020, Zhang *et al.* reported that the expression levels of interleukin-18 (IL-18), cysteine protease-1 (caspase-1), and high mobility group protein B1 (HMGB1) transcripts and proteins were elevated in the lung tissues of the MCT-induced PAH model rats; this linked pyroptosis to the upregulation of the programmed death receptor 1 (PD-1) in the human PASMCs 7. In 2021, Zhang and Liu performed transcriptome analysis of the lung tissue samples from 15 PAH patients and 11 normal controls, and identified 514 differentially expressed genes (DEG) based on the Wilcoxon rank sum test and weighted gene correlation network analysis. Subsequently, they used the FerrDb database and identified eight ferroptosis-related DEGs; this demonstrated that ferroptosis was upregulated in the lung tissues during PAH and was a potential therapeutic target 8. In 2021, Lv *et al.* analyzed blood samples from 50 healthy controls and 88 patients with PAH, including those with idiopathic pulmonary hypertension (IPAH), chronic obstructive pulmonary disease-associated pulmonary hypertension (COPD-PH) and chronic thromboembolic pulmonary hypertension (CTEPH), and reported the association of PAH pathogenesis with the upregulation of parthanatos, a poly-ADP ribose polymerase 1 (PARP1)-dependent and apoptosis inducing factor (AIF)-mediated caspase-independent cell death mechanism; higher PARP1 and AIF expression levels were associated with increased morbidity and mortality of PAH patients 9 (Figure 1).

## Cell death mechanisms in PAH

### Apoptosis and PAH

Apoptosis is a programmed cell death mechanism that plays a crucial role in development and tissue homeostasis by removing abnormal cells in the multicellular organisms 10. Apoptosis is a complex process regulated by multiple genes, including Bcl-2 and caspase family of proteins, oncogenes, and tumor suppressor genes 11,12. The dysregulation of apoptosis is linked to various human diseases, including PAH.

Tissue injury and hypoxia promotes pulmonary vascular remodeling and PAH development by disrupting the balance between cell survival and apoptosis 13.

Several studies have demonstrated that apoptotic PAECs play a critical role in the pathogenesis of PAH. Many diseases and environmental factors induce pulmonary vascular endothelial injury by enhancing endothelial cell apoptosis and dysfunction. Subsequently, increased proliferation of the smooth muscle cells and fibroblasts promote pulmonary arterial remodeling and thrombosis. Furthermore, upregulated apoptosis of PAECs causes severe PAH and triggers excessive proliferation of apoptosis-resistant endothelial cells and subsequent formation of plexiform lesions and pulmonary vascular occlusions 13. Therefore, inhibition of PAEC apoptosis at various stages of PAH development represents a novel therapeutic strategy. In general, the rates of endothelial cell proliferation and apoptosis rates are very low. The dynamic balance between proliferation and apoptosis is required for maintaining a constant number of endothelial cells and normal vascular function. Endothelial injury in the pulmonary precapillary arterioles serves as the initial trigger for PAH pathogenesis, whereas excessive apoptosis of the endothelial cells is a key mechanism of vascular endothelial injury and dysfunction 14. Pulmonary vascular remodeling, plexiform lesions, and *in-situ* thrombosis represent the primary pathological changes in response to aberrant endothelial cell apoptosis in the pulmonary vascular system 15. PAECs are localized in a stable internal environment. However, genetic mutations in the endothelial cells and other extrinsic factors induce endothelial cell apoptosis and pulmonary vascular endothelial damage in the genetically susceptible individuals that are exposed to environmental factors such as viruses, inflammation, hypoxia, blood flow shear force, or certain drugs 16,17. Taraseviciene-Stewart *et al.* reported severe PAH in rats treated with chronic hypoxia and vascular endothelial growth factor (VEGF) receptor blocker SU-5416; the PAH model rats showed significant PAEC apoptosis in the early stages followed by precapillary arterial occlusion because of endothelial cell hyper proliferation; monocrotaline-induced PAH model also confirmed that endothelial cell apoptosis played a significant role in the pathogenesis of PAH 18,19. These results demonstrated that apoptosis of PAECs was an important early event in PAH pathogenesis.

PAH is characterized by elevated peripheral pulmonary vascular resistance because of vascular tension, smooth muscle cell contraction, and vascular remodeling. The hypertrophy, proliferation, migration, and anti-apoptotic properties of the PASMCs are key factors that contribute to these vascular changes 20. Increased activation of the anti-apoptotic mechanisms in the PASMCs promote abnormal smooth muscle cell proliferation and pulmonary vascular remodeling, which is histologically manifested by increased number of PASMCs, disordered arrangement, thickening of the media, significant narrowing of the peripheral pulmonary artery lumen, and complete occlusion of pulmonary arterioles in the advanced stages of PAH. Functionally, impaired regulation of vascular tension, decreased diastolic ability, increased systolic sensitivity, and dysfunction of the systolic and diastolic phases lead to a persistent increase in the pulmonary vascular resistance and mean pulmonary artery pressure 21,22. Therefore, inhibition of anti-apoptotic mechanisms, including suppression of Bcl2 expression delays disease progression 23. Furthermore, inhibition of myelin and lymphocyte protein (MAL) suppressed hypoxia-induced PASMCs proliferation and promoted apoptosis 24. Moreover, pro-apoptotic proteins such as Bcl-xL induced PASMCs apoptosis and reversed pulmonary vascular remodeling 25,26. The delicate balance between proliferation and apoptosis of the PASMCs plays a crucial role in the pathogenic pulmonary vascular remodeling underlying PAH. Therefore, targeted inhibition of PASMCs and/or induction of PASMCs apoptosis represents a novel therapeutic approach to reverse pulmonary vascular remodeling and manage PAH.

Several genetic factors have been associated with an increased risk of pulmonary arterial hypertension (PAH). Functional mutations in the *bone morphogenetic protein receptor 2* (*BMPR2*) gene are commonly associated with an increased risk of PAH. Germline mutations in *BMPR2* are found in 70% of cases with familial PAH (FPAH) and 10% to 30% of cases with idiopathic PAH (IPAH) 27. Research showed that puerarin administration exerted significant protective effects in both hypoxia and MCT-induced experimental PAH rodent models, evidenced by significantly reduced right ventricular systolic pressure (RVSP) and lung injury, improved pulmonary artery blood flow as well as pulmonary vasodilation and contraction function, inhibited inflammatory responses in lung tissues, improved resistance to apoptosis and abnormal proliferation in lung tissues, attenuated right ventricular injury and remodeling, and maintained normal function of the right ventricle. Revealed that MCT and hypoxia treatment significantly downregulated BMPR2/Smad signaling in the lung tissues and PPARγ/PI3K/Akt signaling in the lung tissues and right ventricles, which were restored by puerarin administration 28. BMPR2 mediates anti-apoptotic effects in the endothelial cells. Genetic deletion of endothelial BMPR2 promotes PAH by enhancing endothelial cell apoptosis 29. BMP2 also mediates anti-proliferative and pro-apoptotic effects in the pulmonary arterial smooth muscle cells (PASMCs) by modulating the expression and function of the voltage-gated K^+^ channels 30-31.

In the pulmonary vascular cells, mitochondria play a crucial role in maintaining energy balance and regulation of cell death 32. The dynamic balance between fusion and fission of mitochondria plays a key role in cellular apoptosis 33. Increased mitochondrial fission is linked with elevated apoptosis in pathological conditions such as pulmonary arterial hypertension (PAH). In PAH, upregulation of mitochondrial fission proteins such as MiD and Drp1 accelerate mitosis in the PASMCs and reduce apoptosis. Conversely, silencing of MiD49 or MiD51 promotes apoptosis by restoring mitochondrial fusion, increasing Bak expression, and reducing Akt activation 34. The epigenetic regulation of mitochondrial dynamics contributes to apoptosis resistance in both the human and experimental animal and cellular models of PAH 35,36.

The Warburg effect plays a significant role in the development of pulmonary arterial hypertension (PAH) by promoting excessive cellular proliferation and apoptosis resistance 37. Mitochondria in the PASMCs act as oxygen sensors and trigger hypoxic pulmonary vasoconstriction under low oxygen concentrations. Acquired mitochondrial abnormalities, including elevated expression and activities of pyruvate dehydrogenase kinase (PDK) and pyruvate kinase muscle subtype 2 (PKM2) promote uncoupled glycolysis (Warburg effect), which is linked with PAH development 35. The glycolysis inhibitor 3-bromopyruvate (3-BrPA) inhibits the mitochondria-localized hexokinase-2 (HK-2) and promotes activation of the apoptotic pathway through the release of cytochrome C into the cytosol and activation of caspase-3 (Casp-3) 38. Furthermore, 3-BrPA reversed hypoxia-induced PAH in the monocrotaline (MCT)-induced PAH model rats by inhibiting glycolysis and significantly reducing various pathological processes associated with PAH development 39. This suggested that 3-BrPA exerted its beneficial effects on PAH by specifically targeting HK-2 to induce apoptosis and inhibit inflammation. Therefore, 3-BrPA is a promising therapeutic option for PAH. Furthermore, reduced oxidative phosphorylation and increased glycolysis in the endothelial cells and smooth muscle cells decreases the threshold for apoptosis activation by lowering the levels of mitochondrial reactive oxygen species (ROS) and increasing the mitochondrial membrane potential 40. Therefore, a deeper understanding of the molecular mechanisms governing mitochondrial metabolism and dynamics could reveal new biomarkers and therapeutic targets for PAH.

**Figure 2:**
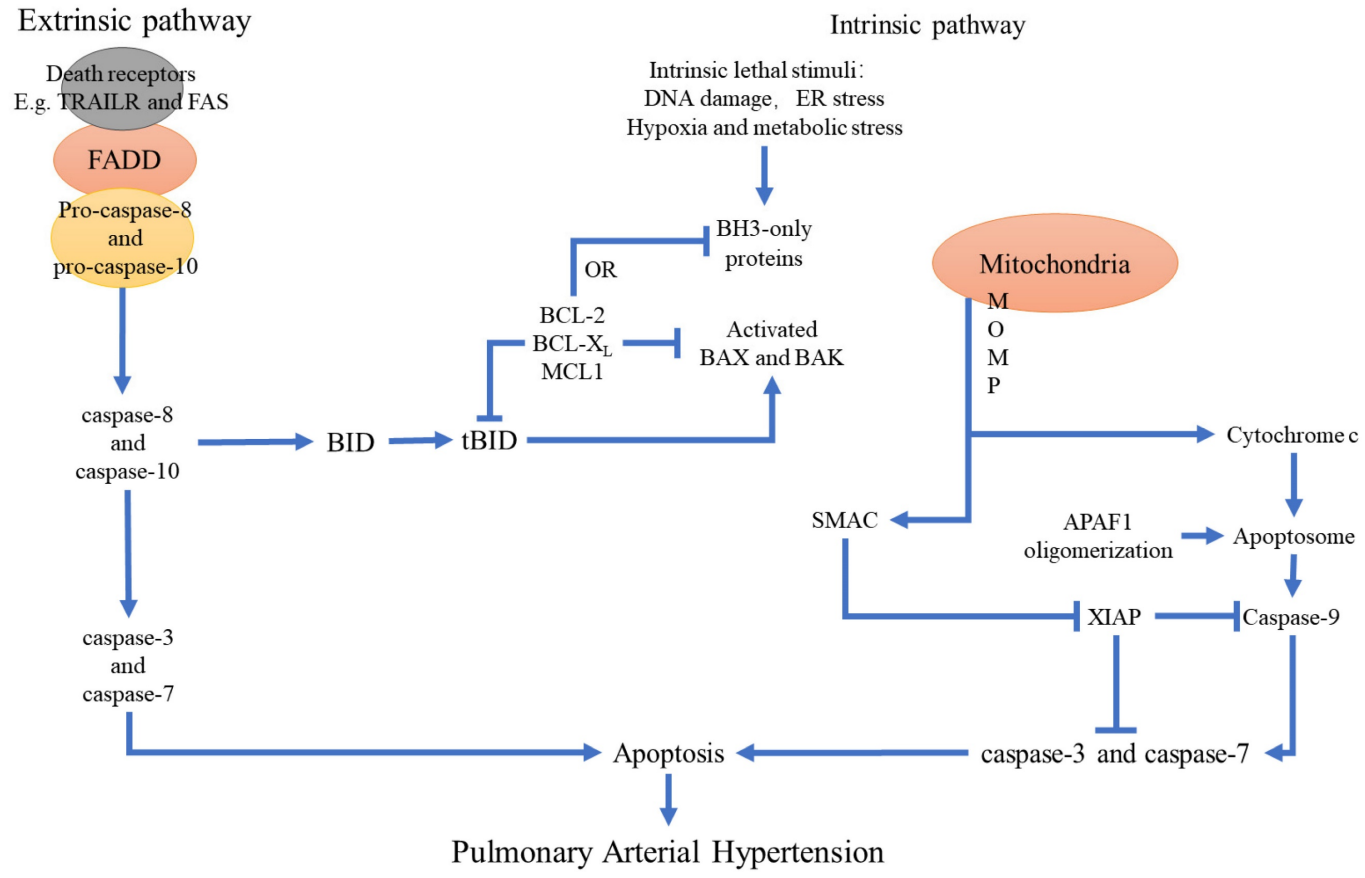
** The relationship between apoptosis and the main pathological changes in PAH.** Apoptosis plays a key role in pulmonary hypertension. Stimulation of various apoptotic or pro-apoptotic factors because of injury and/or hypoxia promotes changes in the structure and function of the pulmonary artery wall and generates an imbalance between cell survival and apoptosis. This leads to pulmonary vascular remodeling and eventually PAH.

The result of abnormal extracellular signaling is the activation of anti-apoptotic cell signaling pathways, such as AKT, ERK1/2, NF-κB and STAT3 41-44, and the inactivation of the pro-apoptotic FOXO signaling pathway 45. In the hypoxia and MCT-induced experimental models of PAH, ERK1/2 and AKT pathways play a key role in the initiation of apoptosis by regulating the expression levels of Bcl2 and the proteins of the caspase family 46,47. Furthermore, in the hypoxia-induced PAH models, cIAP and BCL2 levels are increased and the levels of BAX and BIM are decreased in the endothelial cells and the smooth muscle cells. Moreover, inhibition of the PI3K/AKT pathway, anti-apoptotic BCL2 protein, and cIAP, as well as activation of FOXO signaling alleviates PAH in the animal models 48. Loss-of-function mutations in *BMPR2* promote apoptosis resistance in the smooth muscle cells through the STAT3 signaling pathways 30. These findings demonstrate that targeting the apoptosis and anti-apoptotic pathways in the pulmonary vascular cells may potentially abrogate PAH progression and offer novel avenues for preventing PAH (Figure 2).

### Autophagy and PAH

Autophagy is a type of programmed cell death that is required for maintaining tissue homeostasis and involves a process of “self-digestion”, which is characterized by the engulfment and degradation of damaged cells, organelles, proteins, and pathogens through the lysosomal system 49. Autophagy is categorized into the following three types based on the mechanisms by which the intracellular materials are transported to the lysosomes: chaperone-mediated autophagy, microautophagy, and macroautophagy 50. Aberrant up- or down-regulation of autophagy is implicated in several human diseases such as PAH 51.

Autophagy dysregulation in the PASMCs and PAECs is implicated in pulmonary vascular remodeling and the pathogenesis of PAH 52. For example, estradiol directly inhibits vascular endothelial cell proliferation, improves hemodynamics, and mitigates PAH development by enhancing autophagy, especially mitophagy 53. Beclin-1 knockdown showed defective autophagy and increased endothelial angiogenesis in the pulmonary artery 54. Moreover, in HIV-related PAH, autophagy promotes transition of PAECs from an apoptotic phenotype to a hyperproliferative phenotype 55. This suggested a close relationship between the onset of PAH and increased autophagy in the pulmonary endothelium. Therefore, autophagy inhibition is a promising therapeutic option for the PAH patients, but further validation is required through basic and clinical research.

In the monocrotaline (MCT)-induced PAH model, inhibition of autophagy in the PASMCs suppressed lysosomal degradation of bone morphogenetic protein receptor type 2, downregulated the DNA-binding inhibitory factor 1 (Id1), and suppressed the progression of PAH 51.

Conversely, MCT treatment increased autophagy in the PASMCs and was associated with significantly higher PASMCs proliferation and migration, pulmonary artery remodeling, and development of PAH 51. Moreover, increased expression of LC3B and Beclin1 in the hypoxia-induced PAH models suggested that autophagy played a significant role in PAH pathogenesis 56. The induction of autophagy is a multifaceted process and is influenced by various extrinsic and intrinsic factors such as drugs, cigarette smoke extracts, and mitochondrial dysfunction 57-58. The regulatory mechanisms of autophagy vary in the PAH model rats and include the AMPK-mediated mTOR signaling pathway, BNIP3-dependent Beclin-1 signaling pathway, and the NF-κB signaling pathway 59. Therefore, targeted drugs to inhibit these autophagy-related signaling pathways are promising avenues for treating human diseases with enhanced autophagy, including PAH.

**Figure 3:**
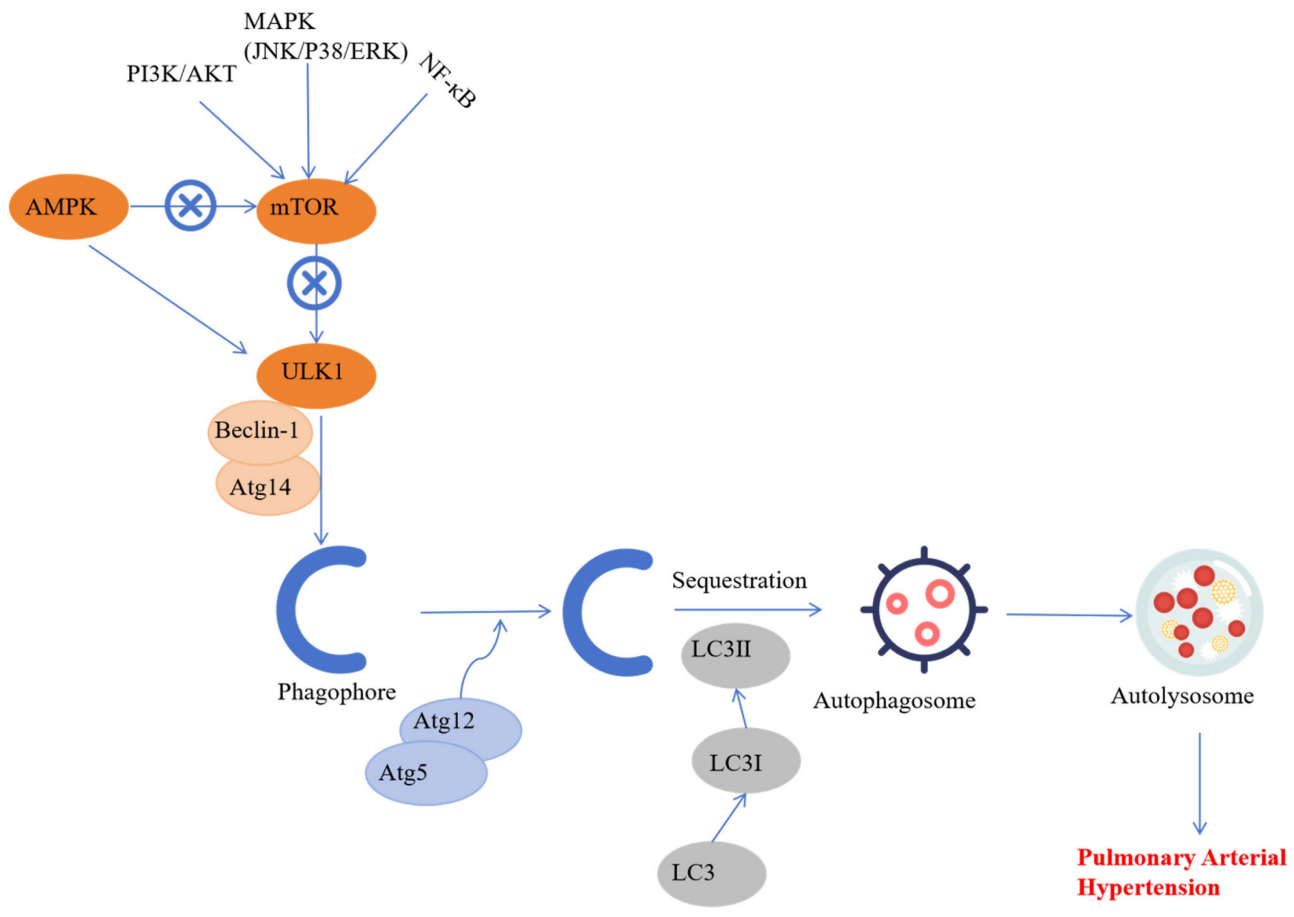
** The role of autophagy in the pathology underlying pulmonary artery hypertension.** Autophagy is activated in several cell types of the pulmonary artery during PAH through multiple signaling pathways. Although several autophagy pathways related to pulmonary hypertension have been reported, the precise molecular mechanism that regulates dysfunctional autophagy during PAH development and progression remains to be determined.

These findings indicate that autophagy is activated in various cell types during PAH through multiple signaling pathways. Although several signaling pathways related to PAH-induced autophagy have been identified, the precise molecular mechanism remains unclear. Therefore, further research is required to determine the molecular mechanisms that regulate PAH-associated autophagy (Figure 3).

### Pyroptosis and PAH

Pyroptosis is a novel type of inflammation-activated programmed cell death during which inflammasome sensors in the human body recognize pathogenic signals and damage-related molecules related and activate caspase-1/4/5/11. Subsequently, gasdermin-D and other family members of the gasdermin family of proteins are cleaved by the activated caspase-1/4/5/11into a N-terminal pore-forming domain (PFD) and a C-terminal repressor domain (RD). PFD induces cell swelling and rupture by generating macropores in the cell membrane 60. Several studies have demonstrated that pyroptosis plays a key role in the development of PAH and inhibition of pyroptosis reduces the severity of PAH 61-63.

Activation of inflammatory caspase is a critical event in the initiation of pyroptosis. Caspase-1/4/5/11 plays a significant role in the development of PAH 61. NLRP3, a prominent inflammasome, triggers pyroptosis in the pulmonary arterial endothelial cells by generating activated pro-inflammatory cytokines, which subsequently promote PAH progression 62. The classic caspase-1-related pyroptosis pathway involves cytosolic pattern-recognition receptors (PRRs) that recognize pathogen-associated molecular patterns (PAMPs) and damage-associated molecular patterns (DAMPs) and assemble ASC and pro-caspase-1 to form functional inflammasome complex; pro-caspase-1 is then cleaved to generate the activated cleaved caspase-1, which cleaves gasdermin-D to generate PFD; pores generated by PFD in the cell membrane stimulate the release of activated pro-inflammatory factors 63. The inflammation promotes vascular remodeling and disease progression in PAH. Inhibition of caspase-1-mediated canonical inflammasomes mitigates PAH 64. Circ-Calm4 is a specific circular RNA that modulates pyroptosis-related signaling pathways and targets in the PASMCs, thereby regulating PAH; moreover, inhibition of circ-Calm4 significantly reduced the levels of pyroptosis-related proteins such as NLRP3, caspase-1, IL-1β, and IL-18 in the PASMCs 65.

The non-classical pyroptosis pathway is mediated by direct binding of caspase-4/5/11 to the lipopolysaccharides and subsequent cleavage of gasdermin-D to form the pore-forming domain (PFD) and release of the activated pro-inflammatory factors. This process mediates endothelial dysfunction and leads to enhanced vascular inflammation, vascular remodeling, and right ventricular failure, thereby contributing to the pathogenesis of PAH 63. Endothelial dysfunction in PAH is triggered by the tumor necrosis factor-α (TNF-α), which is elevated in patients with PAH and the animal models of PAH. TNF-α stimulation mitigated PAH development by inhibiting caspase-4/5/11 66. Moreover, pyroptosis involved direct binding of the caspase-4/5/11 to capsase-3 and activation of the caspase-3-GSDME axis; cleavage of GSDM-E by caspase-3 induced pyroptosis 66. Therefore, inhibition of caspase-3 expression is another potential therapeutic strategy to prevent PAH development.

Caspase-8 promotes synthesis and processing of pro-IL-1β through the non-classical and classical pathways by regulating the formation of the functional NLRP3 inflammasome and cleaved caspase-1. Experiments in the caspase-8 gene knockout SD rats and mice demonstrated that caspase-8 was required for the secretion of IL-1β via the NLRP3 inflammasome and caspase-1 pathways in the macrophages. Subsequent proliferation of the PASMCs and infiltration of the inflammatory cells promoted development of PAH. However, in the Caspase-8 knockout rats and mice, the progression of PAH was inhibited 62.

**Figure 4:**
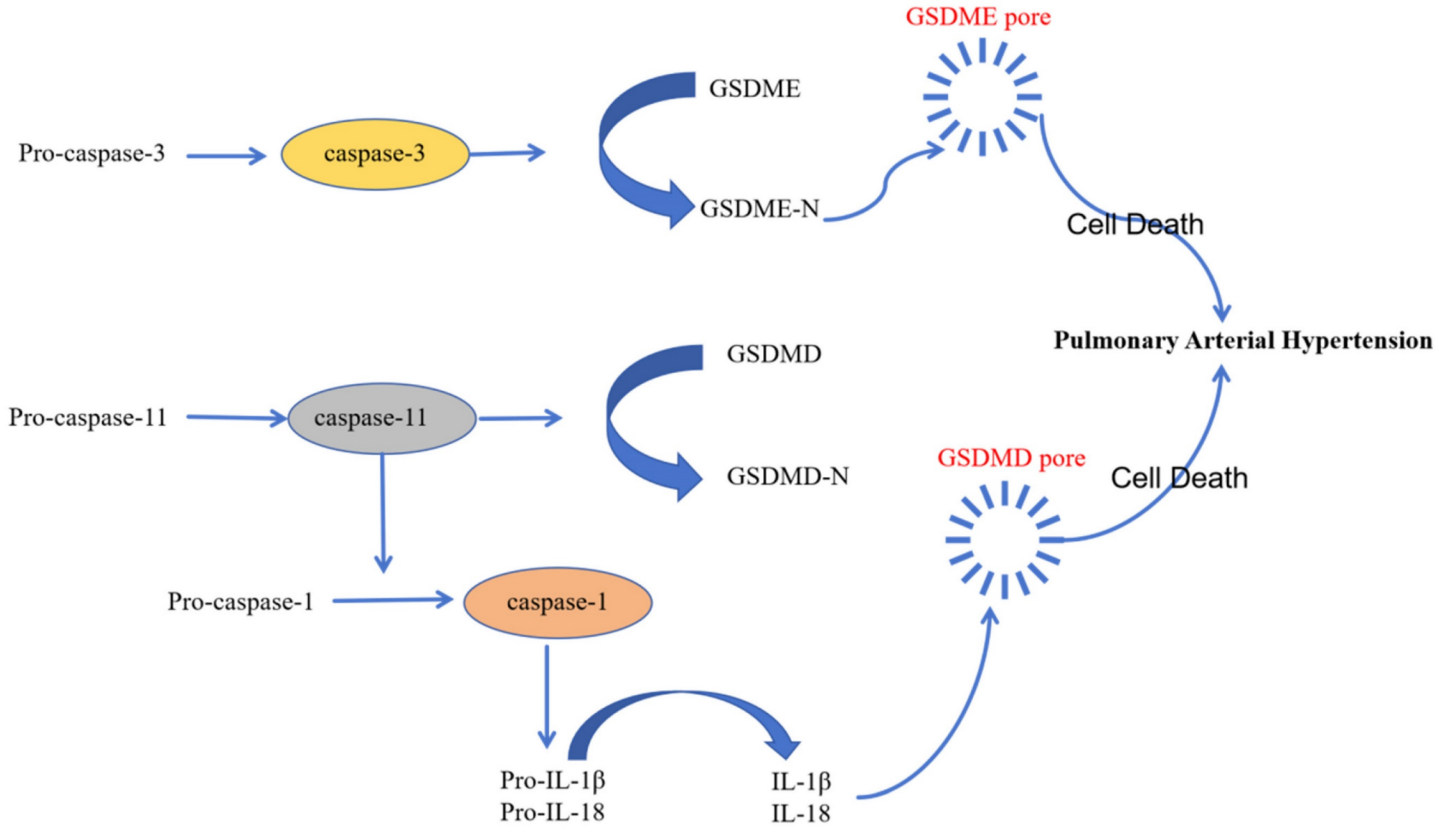
** The role of pyroptosis in the pathology of PAH.** Pyroptosis plays an significant role in the pathogenesis of PAH. The occurrence and development of pulmonary hypertension is mediated by the classical caspase-1 pathway, non-classical caspase-4/5/11 pathway, and the caspase-3-GSDME pathway of pyroptosis.

The diverse recognition of various pathogens and injury-related molecular patterns by the inflammatory sensors demonstrates the pivotal role of pyroptosis in the body's defense against various pathogens. Pyroptosis initiates release of cellular contents from infected cells. This acts as a signal for initiating the inflammatory cascade. The localized inflammation promotes recruitment and activation of immune cells, which assist in the elimination of pathogens from the human body. Therefore, investigating the relationship between pyroptosis and PAH may unravel novel signaling components and pathway mechanisms, thereby revealing new avenues for the prevention and treatment of PAH (Figure 4).

### Ferroptosis and PAH

Ferroptosis is a novel form of non-apoptotic cell death that is dependent on iron and reactive oxygen species (ROS) and is characterized by a reduction in cell size and thickening of the mitochondrial membrane 67. During ferroptosis, excessive accumulation of iron in the cells generates excessive reactive oxygen species (ROS), which leads to the oxidation of polyunsaturated fatty acids, damage to the cell membrane structure, and cell death 68. Aberrant changes in ferroptosis are associated with PAH. Ferroptosis is upregulated in the pulmonary arterial endothelial cells (PAECs) of the MCT-induced PAH rat models, and is characterized by lipid peroxidation, increased cellular iron levels, mitochondrial damage, abnormal expression of GPX4, ferritin heavy chain 1 (FTH1), and NADPAH oxidase 4 (NOX4), activation of inflammatory factors, and severe pulmonary artery remodeling. Furthermore, inhibition of ferrostatin-1 (an iron death inhibitor) delayed pulmonary vascular remodeling and protected right ventricular function in the PAH patients 67. Moreover, PRDX6 is an important driver of PAH progression by mediating ferroptosis in the PAECs through the release of HMGB1 and activation of the TLR4/NLRP3 inflammatory signaling pathway 69. PASMCs are another key target cell for ferroptosis in PAH. Solute carrier family 7 member 11 (SLC7A11) is up-regulated in the Sugen5416/hypoxia-induced PAH rats and the PAH patients. Overexpression of SLC7A11 inhibits ferroptosis and promotes PASMC proliferation 70. Proteomic analysis showed that the ferroptosis pathway was enriched in patients with severe PAH. Furthermore, the presence of the SNP rs1444732 in GPX4 was associated with severe PAH 71. These findings demonstrate that ferroptosis played a key role in the pathogenesis of PAH. Therefore, ferroptosis inhibitors or targeted drugs against ferroptosis-regulatory genes are potential therapeutic targets in PAH. However, further basic and clinical studies are necessary.

### Parthanatos and PAH

Parthanatos is a form of programmed cell death that is initiated by the hyperactivation of poly(ADP-ribose) polymerase 1 (PARP-1) and downstream events involving activation of the apoptosis-inducing factor (AIF), DNA fragmentation, and cell death 72. This process is independent of the caspases and Bcl-2, and is characterized by mitochondrial dysfunction, excessive generation of reactive oxygen species (ROS), and alterations in the calcium channel activity., Parthanatos is characterized rupture of the plasma membrane but does not involve formation of apoptotic bodies and cell swelling as observed during apoptosis 73. The Parthanatos cascade involves four main steps: (1) poly-ADP ribose polymer (PAR) accumulation because of hyperactivation of PARP-1; (2) subsequent release of AIF from mitochondria due to excess PAR; (3) binding of AIF with MIF and translocation of the AIF/MIF complex to the nucleus; and (4) MIF-induced DNA fragmentation in the nucleus 74,75. Parthanatos is triggered not only by severe DNA damage but also by oxidative stress, hypoxia, hypoglycemia, and inflammation 76. A previous study demonstrated that treatment of rats with MCT and Su5416/hypoxia (SuHx) enhanced right ventricular myocardial apoptosis through various pathways and subsequently lead to PAH 77. A recent clinical study reported that circulating levels of PARP1, PAR, AIF, and MIF were higher in the non-surviving patients with PAH compared with the surviving patients with PAH. The plasma levels of PARP1, PAR, AIF, and MIF correlated significantly with the mean pulmonary arterial pressure (mPAP), mean right atrial pressure (mRAP), pulmonary vascular resistance (PVR), and the cardiac index (CI) of the PAH subjects. Elevated levels of PARP-1 and AIF were associated with morbidity and mortality of PAH patients and are considered as strong predictors of increased PAH risk 9. These data demonstrated that the key molecules involved in the Parthanatos pathway, including PARP-1, AIF, and MIF are potential therapeutic targets for PAH.

### Anoikis and PAH

Anoikis is a form of apoptotic cell death caused by detachment of cells from the extracellular matrix because of disruptions in the integrin connections 78. Integrins are cell adhesion receptor proteins that mediate intercellular interactions as well as interactions between the extracellular matrix and proteins of cell cytoskeleton such as connexins. Integrins regulate cell-cell adhesion and transmit important intracellular signals for cell survival, proliferation, gap junction and motility, and are critical determinants of donor cell survival in case of stem cell transplantation 79,80.

Stem cells have emerged as promising candidates for the treatment of patients with PAH because of their ability to self-renew and differentiate into multiple cell types. However, the low survival rate of the transplanted stem cells (typically less than 1%) has significantly hindered the success of stem cell therapy 81. The low survival and engraftment rates of stem cells post-transplantation are primarily attributed to the activation of anoikis, a novel mechanism of cell death 82-85. Therefore, enhancing the adhesion properties of the stem cells at the injury site is critical for promoting their survival and engraftment, as well as, suppressing the activation of anoikis. Previous studies have shown that the levels of integrin α5 and integrin β1 are significantly reduced in the pulmonary arterial smooth muscle cells (PASMC) during PAH 86. Moreover, integrins facilitate endothelium-dependent vasodilation through the nitric oxide (NO) pathway 87. Therefore, NO is a promising treatment for PAH because it can increase the resistance against anoikis and improve cell migration 88-90. Chen *et al.* transfected recombinant bone marrow mesenchymal stem cells (rBMSCs) with a lentiviral vector encoding ITGA5B1 and reported that the recombinant BMSCs with ITGA5B1 showed enhanced cell adhesion, viability, and NO production and reduced anoikis 6,86. Since the levels of ITGA5B are reduced in PAH, the transgenic expression of ITGA5B in the MSCs may offer a promising therapeutic strategy to alleviate PAH in the future.

### Cell senescence and PAH

Cell senescence is an irreversible cell cycle arrest caused by a variety of physiological and pathological stressors, including reactive oxygen species, DNA damage, active oncogenes, and metabolic stress 91. The senescent cells do not proliferate but have metabolic activity and showing characteristic morphological and physiological features, including enlarged flat shape, accumulation of β-galactosidase, and a secretory phenotype. The senescent cells regulate the tissue micro-environment in a paracrine manner. In general, tissue remodeling after transient induction of senescence is beneficial for eliminating damaged cells 92. However, prolonged senescence or inability to eliminate the senescent cells is harmful. The concept of cell senescence was first reported by Hayflick and Moorhead in 1961 based on a permanent loss of cell division in the normal human embryonic lung fibroblasts *in vitro*; similar effects were later confirmed in various other cell types 93-95. The involvement of senescence in lung diseases was first reported in patients with chronic obstructive pulmonary disease (COPD), especially those with emphysema 96. Subsequently, senescent pulmonary artery smooth muscle cells were reported in patients with COPD-PH 97. More recent evidence has suggested that the presence of senescent pulmonary artery smooth muscle cells and endothelial cells in patients with pulmonary arterial hypertension (PAH) is associated with pulmonary artery remodeling and PAH development 98.

The senescence of PASMCs plays a crucial role in the pathogenesis of PAH. PASMCs are key components of the pulmonary vasculature and are responsible for regulating vascular tone and maintaining a normal contractile function of the blood vessels. Extrinsic factors such as smoke, dust, hypoxia, and bacteria, as well as intrinsic factors such as inflammation and cellular stress accelerate the senescence of PASMCs. The senescent PASMCs exhibit enlarged and flattened cell morphology, expression of senescence-associated β-galactosidase, osteopontin, and cyclin-dependent kinase inhibitors, and telomere shortening 97,99. In the hypoxia-treated mice and monocrotaline-induced PAH rats, p53 expression is reduced in the aging PASMCs and may contribute to pulmonary vascular remodeling by promoting PASMC proliferation and calcium influx 100. Furthermore, PASMCs from idiopathic PAH patients show reduced expression levels of the p53 protein and increased Bax/Bcl-2 ratio 101,102. This suggested that PASMC senescence was a critical factor in the process of pulmonary vascular remodeling during PAH. Therefore, targeting p53 is a potential therapeutic strategy to prevent PASMC senescence and subsequent alleviation of PAH.

PAEC senescence plays a significant role in the development of PAH. The levels of senescence markers in the PAECs are significantly higher in the patients with idiopathic PAH (IPAH) compared to those in the healthy subjects. The expression of TWIST1 and PDGFB is elevated in the senescent cells and mediates the progression of PAH 103. DNA damage and senescence is induced by shear stress in the pulmonary microvascular endothelial cells (MVECs) of patients with idiopathic PAH 104. Furthermore, telomere erosion is associated with a higher proportion of senescent lung endothelial cells in patients with chronic obstructive pulmonary disease (COPD) 105. Hypoxic mice with endothelial FXN deficiency exhibit cell-specific senescence, elevated expression of the senescence-associated secretory phenotype (SASP) markers, accumulation of perivascular monocytes, and increased vascular collagen deposition. This suggested that endothelial FXN deficiency exacerbated endothelial cell senescence and worsened the severity of PAH 106,107. Collectively, these findings suggested a close association between endothelial cell senescence and the development of PAH. Therefore, targeting senescence and its associated proteins are potential therapeutic targets for PAH in the future.

Further investigations are necessary to unravel the role of cellular senescence in the pathophysiology of PAH and for preclinical trials regarding the efficacy of senescence-targeting drugs. Although there is a great deal of evidence regarding the close association of senescence in the PASMC and PAECs with PAH, it is not clear whether these cells influence the development of PAH together or independently. Secondly, the occurrence of PAH is multifactorial, and the relationship between senescence and other pathogenic factors is not completely clear. The clinical importance of senescence is a treatment target requires in-depth study. Furthermore, the study on the molecular mechanisms underlying the role of cell senescence in PAH is still in the preliminary stages. Therefore, further basic and clinical research is necessary. It is plausible that once the integral role of cell senescence has been confirmed in PAH, inhibition of senescence or the removal of senescent cells will provide a novel avenue for treating patients with PAH.

## Summary and Future Perspectives

In the pathogenesis of pulmonary arterial hypertension (PAH), the cells of the pulmonary arterial system undergo programmed cell death through different pathways. However, there is a significant lack of information regarding the relationship between different mechanisms of programmed cell death and PAH pathogenesis. Therefore, significant investigations are necessary to elucidate the mechanisms by which different cell death mechanisms are triggered *in vivo*. Moreover, further research is necessary to determine how these survival and death signals are balanced to determine specific cell fate. It is also not clear as to how these complex signals in the dying cells influence PAH development and progression. This remains a promising area of research in the field. The cell death mechanisms and signaling pathways play a critical essential role in growth, development, homeostasis, and disease. Deciphering these mechanisms will unravel novel therapeutic targets for the prevention and alleviation of PAH.

## Figures and Tables

**Table 1:** Multiple types of cell death mechanisms in PAH

Cell death mechanisms	Stimulation	Features	Morphology	Molecular mechanisms
Apoptosis	Physiology or pathology	Absence of inflammatory response; formation of apoptotic bodies	Chromatin aggregates, divides, localizes to the nuclear membrane; cytoplasm condenses; finally the nuclear membrane ruptures; cells form many apoptotic bodies by budding.	AKT, ERK1/2, NF-κB and STAT3 signaling pathways; imbalance of mitochondrial dynamics imbalance; Warburg effect.
Autophagy	Physiology or pathology	Autophagosome formation	The autophagosome is a vacuole-like structure with double or multilayer membrane and contains cytoplasmic components, including mitochondria, endoplasmic reticulum, and ribosomes.	AMPK-mediated mTOR autophagy signaling pathway; BNIP3-dependent Beclin-1 autophagy signaling pathway; NF- κ B signaling pathway
Pyroptosis	Pathological stimulation	inflammatory reaction; pyroptotic body formation	Under electron microscope, rupture of the plasma membrane is clearly seen; large number of vesicles or pyroptotic bodies seen; pores are formed in the cell membrane; the cell membrane bursts and releases the intracellular contents.	Non-Classic caspase--4/-5/-11 signaling pathway;Classic caspase-1signaling pathwayand GSDMD/GSDME signaling pathway
Ferroptosis	Pathological stimulation	Intracellular accumulation of iron ions; lipid peroxidation; elevated ROS.	Cell membrane rupture and blistering; mitochondrial atrophy; decrease or even disappearance of the mitochondrial ridge; increased membrane density; normal nuclear morphology; lack of chromatin condensation	HMGB1-mediatedsignaling pathway; TLR4/NLRP3signaling pathway
Parthanatos	Pathological stimulation	Activated PARP1 binds to AIF/M1 and mediates the translocation of AIF/M1 complex from the mitochondria to the nucleus; MI stimulates DNA fragmentation.	Chromatin condensation; DNA fragmentation (large)	AIF/M1 dependent and AIF/M1 independent signaling pathway
Anoikis	Pathological stimulation	It only occurs when adherent cells lose their adhesion	Cell volume is reduced; organelles shrink; formation of apoptotic bodies.	Intrinsic pathway (mitochondrial events induced by cell stress) and extrinsic pathway (mediated by TNF and first apoptosis signal (Fas-ligand)
Cell senescence	Physiology or pathology	Cells shrink and become smaller in size; reduced metabolic rate	Cell water content decreases; volume is reduced; cell membrane fragility increases; and permeability decreases	Mechanisms driving cellular senescence include DNA damage, telomere shortening, FXN loss, and elevated SASP

## Abbreviations

AIF: Apoptosis inducing factor
CI: Cardiac index
COPD: Chronic obstructive pulmonary disease
CTEPH: Chronic thromboembolic pulmonary hypertension
DAMP: Damage-associated molecular patterns
DEG: Differentially expressed genes
EPC: Endothelial progenitor cells
IPAH: Idiopathic pulmonary hypertension
MAL: Myelin and lymphocyte
PAEC: Pulmonary artery endothelial cells
PAH: Pulmonary arterial hypertension
PAMP: Pathogen-associated molecular patterns
PAP: Pulmonary artery pressure
PAR: Poly-ADP ribose
PASMC: Pulmonary artery smooth muscle cells
PDK: Pyruvate dehydrogenase kinase
PFD: Pore-forming domain
PRR: Pattern-recognition receptors
PVR: Pulmonary vascular resistance
RD: Repressor domain
ROS: Reactive oxygen species
SASP: Senescence-associated secretory phenotype
VEGF: Vascular endothelial growth factor
